# Practical Tips on Epidermolysis Bullosa for Caregivers: Part 2

**DOI:** 10.7759/cureus.55499

**Published:** 2024-03-04

**Authors:** Aaron Tabor, Jo Ann K LeQuang, Joseph Pergolizzi Jr

**Affiliations:** 1 Research, No Baby Blisters, Colorado Springs, USA; 2 Scientific Communications, NEMA Research, Inc., Naples, USA; 3 Anesthesiology, NEMA Research, Inc., Naples, USA

**Keywords:** rare genetic diseases, pediatric rare diseases, orphan diseases, recessive dystrophic epidermolysis bullosa (rdeb), dystrophic epidermolysis bullosa, epidermolysis bullosa, epidermolysis bullosa simplex, junctional epidermolysis bullosa

## Abstract

The heritable condition epidermolysis bullosa (EB) is a rare but potentially devastating and life-threatening condition that is characterized primarily by cutaneous fragility, manifested when the dermis and epidermis fail to adhere properly. EB has no cure, and because of its rarity, few healthcare professionals have experience in treating it. Most families with an EB child are forced to rely on family caregiving which can be disruptive to family routines but, more importantly, place enormous time and emotional and financial burdens on the family. EB can be extremely painful, and families are often caught in the bind of trying to manage overwhelming financial burdens in an effort to help their children cope with excruciating pain. For many years, the nonprofit organization NoBabyBlisters.org has worked on five continents with families caring for EB children. Many of these families reside in under-developed nations with hot climates and limited healthcare resources. Over time, the healthcare professionals with NoBabyBlisters.org have worked with EB families both internationally and in the United States to develop a series of simple tips or "hacks" that may provide relief or great benefit to these children. The objective of this article is to share these field-tested tips with a wider audience. This is not a scientific study or a systematic review and is offered as a companion article to an earlier article on the same subject.

## Introduction and background

The heritable and heterogenous group of skin disorders characterized by fragile skin and painful blistering, epidermolysis bullosa (EB), has gained increasing attention in the medical and research communities. EB reduces the functional integrity of epidermal adhesion and limits dermo-epidermal anchoring. This manifests as the fragility of the skin with a tendency to induce blisters in the skin and on mucous membranes and pose a risk for infection [1]. While there is no cure, therapeutic advances have explored genetic targets and have also found that reducing the chronic low-grade inflammatory response of EB provides symptomatic relief [2].

A child born with EB can place an overwhelming and unexpected burden on the family, particularly when the more severe forms of EB are involved, some of which may require round-the-clock care [3]. The cost of dressings and other needed supplies can be exorbitant [4]. In fact, in the United States, over a quarter of all families with an EB child in the family stated they spent over $1000 per month out of pocket on wound care alone [5].

Although less frequently discussed, EB can cause the child and the entire family considerable social morbidity in the form of shame or embarrassment, particularly since the disease can be quite visible and most people have no experience with it. EB can cause significant pruritus and severe pain [6]. EB is rare, but global, and many families facing the dilemmas of EB caregiving have limited access to healthcare and low economic resources. Even when EB occurs in the United States or Western Europe, few healthcare professionals have experience in treating it [7].

The ideal treatment paradigm for EB is an interdisciplinary model, but even in the developed world, this does not often occur [8]. Families often find themselves isolated; support, when available at all, often comes via social media channels. The charity NoBabyBlisters.org is sometimes the only organization filling the gap with some families, offering knowledgeable support as well as meeting medical material needs.

EB has a poor prognosis, and life expectancy varies depending on the type and severity of EB. Infections from wounds and squamous cell carcinoma are frequently the cause of death, but many people with EB can live for decades with managed disease provided they receive proper care [6]. There are four main types of EB and many subtypes. The main types are EB simplex, junctional EB, dystrophic EB (the most common form), and Kindler syndrome (the least common form). EB simplex tends to present in the mildest manifestations [7].

The challenges facing caregivers for EB patients as well as the clinicians who treat them are manifold because the condition is irreversible and incurable. This means EB children will require a lifetime of expensive care. However, organizations working with EB patients such as NoBabyBlisters.org have gained valuable field experience employing everyday actionable solutions for EB care. The aim of this article is to present these clinical "hacks" or recommendations for caregivers in light of the fact that there are many small things that can be easily done that may make genuine improvements in the EB patient's situation. This is neither a clinical study nor a systematic review but does present field-tested ideas that may provide relief and comfort to EB families.

## Review

**Figure 1:**
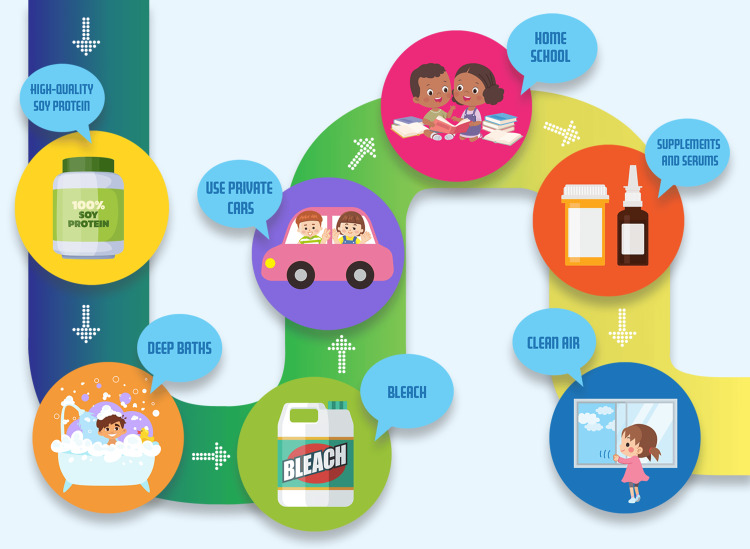
Practical tips developed to help reduce pain and improve the care of children with EB. This is original art by Belinda Kinkade and owned by No Baby Blisters.

NoBabyBlisters.org works directly with EB families on five continents all over the world, providing medical material needs and support, primarily in developing nations with very limited healthcare resources. NoBabyBlisters.org has dual aims: finding a full-body cure for EB and supporting those now trying to manage the care of a family member with EB. The tips presented here were created as a result of real-world experience, and, in that sense, they are all "field tested." These tips emerged and were implemented from observations of EB families by the healthcare professionals who work for NoBabyBlisters.org. These are practical strategies, practiced in some cases among families in very poor nations, that can improve the quality of life in EB patients. See Figure 1.

Eat high-quality soy protein

Soybeans have long been known for their beneficial nutritional content derived from several physiologically active ingredients, including isoflavones. Soy isoflavones have anti-oxidant effects, balance blood sugar, enhance bone density, and are bioactive in the skin [9]. Soy isoflavones can alter dermal connective tissue and help to restore the dermal-epidermal junction [10]. However, it is important to get high-quality soy protein products for optimal benefits. Available as protein powders, they can be easily consumed by people with EB in drinks or smoothies; people with EB may have damaged mucosal tissue or blisters in the esophagus which makes eating solid foods painful. Other soy products include soybeans, tofu, tempeh, soy milk, miso, and edamame.

A deeper dive into the bathtub

The fragile skin of an EB child is vulnerable to shearing, tearing, lacerations, and wounds. Wound care can be the most crucial aspect of EB care since wounds are associated with infections, exudate, and malodor. EB wound care is based on moist wound healing to help encourage epithelialization and reduce the dehydration of fragile tissue [11]. However, the wounds in EB present specific challenges in that they can often be large and may occur in awkward areas of the body, such as under the armpits or around the neck. For optimal healing, the dressings should be securely fixated but not adherent [11]. Dressings are often changed every 24-48 hours, which may involve a prolonged and painful process. Soaking the EB child in a deep bath of warm water can reduce pain and facilitate dressing changes with reduced blood loss. Many EB children are only partially submerged in water during a dressing change, and some caregivers have to use bowls or pots to improvise a non-soaking rinsing solution. In our experience, installing a deep bathtub that allows the child to sit neck-deep in tepid water can be soothing, reduce bleeding, and be less painful than changing bandages "dry" or with minimal water.

Add some bleach

Water and even soaking can be soothing for EB wounds, but treating the water with a small amount of bleach, vinegar, or magnesium salts (Epsom salts) can help kill bacteria and further soothe the angry skin. Bleach is probably the best choice and should be used at a ratio of approximately two teaspoons per 1 gallon of water (one-half teaspoon per liter). The use of bleach in bathwater may increase pruritus, so if the EB child complains about itchiness, it may be preferable to use vinegar or magnesium salts in the water instead of bleach [12]. In a cross-sectional study of 202 respondents with EB, 54% added bleach to cleansing water [13]. While any of the above additives may be beneficial, bleach is the only one with a substantial antimicrobial effect.

Ride in comfort

Even those with severe EB must venture out for special events or medical care. Traveling can be particularly painful if it exposes the person with EB to hard unyielding seats or any type of friction, such as sliding into the seat on a bus. A foam pad or soft cushion can make the ride more comfortable but may make the EB child more conspicuous on public transportation. Taking the bus or other forms of transportation will almost invariably expose the EB child to encounters with people unfamiliar with the disease, who may stare, ask embarrassing questions, or be unkind. Indeed, psychosocial protection may be needed for people with EB, even as they try to go about the normal activities of daily living [14].

Furthermore, in some parts of the world, using public transportation may expose EB children to heat, harsh sunlight, dust, and humidity, which may irritate their delicate skin. EB children may experience urologic accidents or suddenly start to bleed, either of which may be excruciatingly embarrassing for them in a public setting.

Ideally, and since people with EB do not tend to travel much, it is optimal that a private car be used for transportation, even if one has to be borrowed or rented for the occasion. The vehicle should be kept cool, and seats should be padded or cushioned to avoid friction. Taxis may be an alternative although the taxi driver must be cooperative and allow for cushions to be brought into the vehicle and the vehicle temperature to be kept cool. Air conditioning is necessary in warm weather. We recommend that whenever possible, the person with EB should be offered the opportunity to recline or even lie down on the seat rather than sit up, as it may be more comfortable. When driving a person with EB, it is important to avoid sudden stops or abrupt turns that might jostle the passengers.

Home school

While EB children face many struggles, most of them are eager to learn and relish being part of the "real world" beyond EB. The difficulties in allowing an EB child to attend a public or private school can be insurmountable, in part because most schools are not equipped to manage their special needs. Not only are school bathrooms difficult to navigate for children with EB, but urological complications typical of EB may cause debilitating symptoms [15]. Children with EB may experience abrasions or skin damage doing relatively normal things like sitting in wooden chairs, putting arms on the edges of desks, or handling books and notepads. Bleeding events can occur which not only make the child susceptible to potential infection but can also be embarrassing. EB students may spend all or most of their school day in very intense pain. In our experience, most schools in developing countries do not have air conditioning, thus exacerbating EB blisters, itching, and pain. Teachers may not be aware of the nature of EB and might make wrong decisions for the student. EB children cannot participate in sports and many other group activities and therefore feel left out. Other students may bully a child with EB, which can be extremely distressing. Even seemingly benign events such as a hand on a shoulder or a bit of jostling in line can cause excruciating pain for the EB child.

Parents of children with EB should make every reasonable effort to home-school the child. In some parts of the world, there are computer-based homeschool programs that can be used to supplement or direct education [16]. EB children are often very eager to learn, particularly when they can work on tablets or phone apps. NoBabyBlisters.org has provided devices for these children to use with WiFi connections to keep up with their school work. Not only is education valuable, but it allows the EB child to venture beyond the limitations imposed by the disease and distracts them from their painful symptoms.

Supplements and serums

There is no cure for EB, but symptomatic relief may be possible for some patients with certain over-the-counter supplements and serums. For people with EB simplex, the use of sulforaphane can help restore cutaneous integrity by "reprogramming" keratin biosynthesis [17]. Sulforaphane is able to selectively induce certain genes in skin keratinocytes and alleviate blistering in a mouse model. Derived from broccoli sprouts, sulforaphane is an inexpensive serum available over the counter or online. For those with dystrophic EB, kaempferol, a flavonoid derived from plants, can increase the production of mRNA and protein in skin cells [18]. Kaempferol also has antifungal, anticarcinogenic, anti-inflammatory, and antibacterial effects [19]. Like sulforaphane, kaempferol is available over the counter and online.

Home sweet home

The skin is our first line of defense against environmental toxins and pollutants, such as ultraviolet radiation, polycyclic aromatic hydrocarbons, volatile organic compounds, dust and particulate matter, carbon monoxide, ozone, and many other things floating around in the atmosphere [20]. Even healthy skin can be damaged by air pollution. The fragile and frequently inflamed skin of people with EB is particularly vulnerable to pollution [21]. The ancient physician Hippocrates called the therapeutic properties of the ocean "thalassotherapy." In Japan, residing in a marine climate is a type of "climatotherapy," which can be used to treat fatigue, depression, and anxiety [22]. Just inhaling the natural hypertonic saline on an ocean shore can be beneficial, although living in such an area requires extra hydration as ocean air can be dehydrating. If possible, the family should consider moving to such an environment. Even if it is not possible to get home near the coast, the family should try to reside in an area with the least air pollution.

Discussion

The care for persons with EB is palliative, but many EB patients live decades with managed disease. In palliative care, the emphasis is on patient comfort and quality of life, necessitating a holistic view of the person with EB within the context of the disease [23]. Palliation must also include the familial caregivers and the wider family, even extended family, since diseases like EB can have a profound and disruptive effect on the family structure [24]. Caregiving typically falls to a family member, who may become overwhelmed, suffer exhaustion and despair, and experience resentment and anger [25]. As much as possible, caregivers should be offered financial assistance, extra help with caregiving or household chores, and social support, but such aid may not be available, particularly in developing nations or in rural areas [25]. Since EB is a genetic disorder, it occurs sporadically rather than in clusters, so people with EB and those caring for them may be isolated and not know others in the same situation. Online support groups would be beneficial but are few. Charities and other organizations are raising awareness for EB, but this involves slow work.

NoBabyBlisters.org is a charitable organization established with twin goals: to find a cure for EB and to offer support to those families dealing with EB now. To this end, NoBabyBlisters.org delivers monthly medical supplies to families of EB children on five continents. Healthcare professionals who work with and for NoBabyBlisters.org have gained and shared considerable experience in the holistic care of EB patients. Some of the things learned in real-world settings have been that simple tricks or "hacks" can offer great benefits. While dressings and ointments for an EB child may cost a lot, there are numerous simple things that can be done to improve the quality of life of EB patients. These hacks are presented here in an effort to share some of the ways to assist EB patients as work for the cure continues.

These tips to make things easier and more comfortable for people living with EB are described here and in our prior companion article to offer practical and actionable advice. Apart from specialists, most physicians and nurses have no experience with EB. People with EB come from many different regions and backgrounds. Sweden, for example, offers specialized EB services in interdisciplinary clinics that encompass dermatologists, pediatricians, dentists, and other clinicians but recognizes that greater international collaboration may be needed [26]. Furthermore, when non-Swedes come to these clinics for care, there is a language and cultural barrier. Interdisciplinary clinics are a centralized model of care which benefits the local patients; EB on a global scale seems to require a more decentralized approach, such as the one used by NoBabyBlisters.org.

EB is recognized by the medical community and is gaining traction as a research topic [27]. A consensus document was published on wound care in EB in 2012, but the limited research in this field resulted in a document that was based more on expert recommendations and opinions than evidence-based medicine [8]. While it can be tempting to get lost in the big picture of EB, namely, the many urgent needs, such as for curative treatments, greater clinician awareness and training, better symptomatic treatments, scientific studies, and evidence-based guidance, the day-to-day life of people with EB at the moment depends on small steps that can be taken to make life a little more comfortable and to infuse a better quality of life into everyday activities.

This paper has certain limitations. It is a series of recommendations derived primarily from direct experience on the part of clinicians on the front lines of EB treatment. It is not a clinical study or a systematic review of the literature.

## Conclusions

EB is a rare genetic disorder characterized by skin fragility that can be painful, disabling, and even life-threatening. Support for the families of EB children includes medical supplies but also practical advice and simple actionable tips. Some of these tips are shared here, not as evidence from clinical trials but from field-tested experience working with EB children and their families. There are many simple things such as eating high-quality soy protein, bathing in tepid water during dressing changes, homeschooling, and riding in private transportation for excursions that can be highly beneficial.
